# Critique on conclusions regarding toxic compounds in *Jatropha curcas* kernel cake

**DOI:** 10.1038/s42003-021-02869-6

**Published:** 2021-12-01

**Authors:** George Francis, Harinder P. S. Makkar, Reinhold Carle, Martin Mittelbach, Michael Wink, Jorge Martinez Herrera, Rakshit Kodekalra, Klaus Becker

**Affiliations:** 1 Jatropower AG, Haldenstrasse 5, CH-6340 Baar, Switzerland; 2 Adjunct Professor, Center for Agriculture in the Tropics and Subtropics, University of Hohenheim, Stuttgart, Germany; 3 Institute of Food Science and Biotechnology, University of Hohenheim, Stuttgart, Germany; 4 Institute of Chemistry, University of Graz, Graz, Austria; 5 Institute of Pharmacy and Molecular Biotechnology (IPMB), University of Heidelberg, Heidelberg, Germany; 6 Director de Coordinación y Vinculación, del INIFAP en Tabasco, Huimanguillo, Tabasco Mexico; 7 Chief Technology Officer, Medipure Pharmaceuticals, Burnaby, B.C. Canada; 8 Institute for Tropical Agricultural Sciences (Hans Ruthenberg Institute), University of Hohenheim, Stuttgart, Germany

**Keywords:** Biotechnology, Biological techniques, Pathogenesis

**arising from** Wang et al. *Communications Biology* 10.1038/s42003-020-0919-z (2020).

The article from Wang et al.^1^ claims, that *“phorbol esters are absent in jatropha seed cake”*, and draws a conclusion that *“phorbol esters are not responsible for toxicity in conventional jatropha kernel cake”*. In our opinion, such claims and the conclusions are not sufficiently substantiated by the results presented in the paper.

We have analysed some of the claims made in the paper of Wang et al.^1^ in light of previously published information below:

*The main claim is that “For more than a decade, continuous efforts have been taken in our labs to isolate and purify individual phorbol esters from KCakeJ ……. However, neither we nor other researchers have obtained any phorbol esters from KCakeJ”*. Further, it is mentioned *“….that phorbol esters have not been proved to be at detectable level in KCakeJ, it can be concluded that phorbol esters are not the major toxins of KCakeJ.”*

There are several papers that show that phorbol esters have been detected in deoiled Jatropha cake. Faria-Machado et al.^2^ confirmed in their recent paper that phorbol esters were present in the jatropha seed cake that remained after oil extraction. These phorbol esters could be extracted using methanol as solvent. The paper mentions the concentration of up to 1.26 mg phorbol esters/g of jatropha seed cake, measured using the HPLC method (Makkar et al.^3^). They also clearly show the peaks of the different phorbol esters.

Another paper from 2018, published by a Chinese group Li et al.^4^, undoubtedly detected phorbol esters in de-oiled, “detoxified” Jatropha kernel cake obtained from China National Offshore Oil Corporation (CNOOC). The paper mentions that the Jatropha *“cake was detoxified by steam treatment (pre-treatment 80* *°C and 90* *°C until dried) and ethyl alcohol extraction (55* *°C for 2* *h) to remove more PEs (phorbol esters)”*. Even after this detoxification treatment a residual phorbol ester content of 0.11 mg/g of the cake was measured using the HPLC method. The paper by Li et al.^4^ concluded that Jatropha kernel cake was a good protein source for pigs, but the level of phorbol esters should be below 5.5 mg/kg in growing pig diets.

Further Wang et al.^1^ claims that “*Jatropha phorbol esters are widely recognized as the main toxic components of KCakeJ (jatropha seed cake), although direct and convincing evidence is absent*.”

This statement is not substantiated with convincing evidence in the paper. On the contrary, there are several refereed publications that show that phorbol esters are indeed present in Jatropha kernel meal and that they are the toxic components in Jatropha cake and kernel meal.

Becker K and Makkar HPS^5^ have clearly shown that phorbol esters extracted and concentrated from *Jatropha curcas* seed oil using methanol (similar to what was done by Wang et al.^1^) were toxic (feed rejection and presence of excess mucus in the faeces) to common carp (Cyprinus carpio L.) fingerlings when they were added to their diets at a level of 31 µg/g diet.

Devappa et al.^6^ show that phorbol esters can be detected in screw pressed jatropha seed cake and that it is the phorbol esters in the seed cake that are toxic to animals (snails in this case). When the seed cake was mixed in soil, the phorbol esters contained in them were gradually degraded, evidenced by the gradual disappearance of phorbol ester peaks in the chromatogram. The extracts from the soil-jatropha seed cake mixture containing the phorbol esters were initially toxic to snails. After 15 days of incubation the jatropha cake-soil mixture lost toxicity to snails after extracts from this mixture showing much lower peaks of phorbol esters in the chromatogram.

The study done by Kumar et al.^7^ found that the untreated defatted Jatropha kernel meal collected from India had 1.8 mg phorbol esters per g. After complete extraction of the phorbol esters using organic solvents (shown by lack of phorbol ester peaks after extraction through the HPLC method), the kernel meal was used to replace fish meal protein at a level of 50%. The feeding experiment that followed with common carp did not affect growth and nutrient utilisation.

In addition, there exists a non-toxic Jatropha variety in Mexico and the kernels of this variety are locally consumed by humans. The fact that the conventional toxic variety of Jatropha seed kernels differs from the edible, non-toxic variety only in the presence of phorbol esters, is proved by analytical data presented in many publications^8–11^. All other nutritional characteristics and concentration of antinutrients are similar in both toxic and non-toxic varieties of Jatropha.

Also EFSA^12^ (European Food Safety Authority) Panel on Contaminants in the Food Chain (CONTAM) reviewed a vast literature on *Jatropha curcas* and the Panel concluded that phorbol esters present in the Jatropha seed cake are the main toxic agent.

Further, Wang et al.^1^ claims that *“….is based on an assumption that phorbol esters are retained in KCakeJ after oil extraction and on the HPLC “measurements” of phorbol esters using 12-O-tetradecanoylphorbol-13-acetate (TPA) as standard due to the unavailability of Jatropha phorbol esters. However, the chemical structures of Jatropha phorbol esters are quite different from that of TPA. They have a macrocyclic dicarboxylic acid diester structure between the O-13 and O-16 of 12-deoxy-16-hydroxyphorbol”*.

The difference in structures of Jatropha phorbol esters and the standard used (TPA or PMA), is known and discussed in earlier publications, but this standard was still found to be valid for quantification. It is to be mentioned that the phorbol esters are detected based on a characteristic peak structure in the chromatogram when using the HPLC method. Similar peak structure for the PEs in Jatropha seed has been observed and validated by different research groups previously (Makkar et al.^3^, Devappa et al.^13^, He et al.^11^, Baldini et al.^14^, Roach et al.^15^). The standard (TPA/PMA) is used to quantify individual PEs. The individual PEs have also been separately extracted, purified, structure determined, and studied for toxicity as can be seen in previous publications^13,14^.

E.g., the paper by He et al.^11^ mentions: *“…we used phorbol 12-myristate-13-acetate (PMA) as an internal standard to calculate the concentration of the J. curcas phorbol esters. PMA, which is obtained from Croton tiglium, is much simpler than the phorbol esters obtained from J. curcas in that it contains the simple acyl groups acetate and myristate. The UV spectrum of this compound contains a single peak with a lambda max of 242* *nm. The structure of the phorbol esters from J. curcas is more complicated, due to molecular rearrangements which occur resulting in the covalent fusion of the two acyl groups. The UV spectra of these species are therefore more complex, with multiple peaks. Each of the J. curcas phorbol esters contains a peak with lambda max of ca. 280* *nm, which is presumably due to a chromophore that is absent in the simpler PMA. The previous studies using PMA as an internal standard have calculated peak areas at 280* *nm. As PMA does not have a peak with a lambda max of 280* *nm, we instead opted to measure peak areas at 240* *nm.”*.

Further, Baldini et al.^14^ compares the HPLC-UV method with an LC-MS method and also compares the peak of PMA and the peaks of the Jatropha phorbol esters. It also discusses the higher sensitivity of the LC-MS method, but the study also shows the validity of the HPLC method for detecting PEs in Jatropha seeds and leaves. According to them *“The objective of this work was to develop and optimize a LC–MS/MS method for the quantitative determination of PEs (phorbol esters) in seeds and leaves of J. curcas L. plants from Ghana and Mexico and in liver (as an organ with the function of accumulation) from goats fed with PEs in their diet. The HPLC-UV analysis evidenced five chromatographic peaks in the toxic seed kernels corresponding to the factors C1, C2, C3, C6 and C4–C5, respectively, with a PEs concentration of about 5100* *g/g (as TPA/PMA equivalent). No PEs related peaks were detected in Mexican kernel seeds, while in the case of leaves and liver the analysis was hampered by the presence of interfering compounds. The toxic kernel seed extract was used as a standard solution for the PEs quantitation in leaves and liver samples by LC–MS/MS, with the standard addition method. The LC–MS/MS method with a LOD and a LOQ of 0.07 and 0.21* *g/g, respectively, resulted in higher sensitivity and selectivity compared to the HPLC-UV method. All three MRM transitions were present in Ghanaian toxic kernel seed, while no peaks were present in the supposed non-toxic Mexican kernel seed.”*

Wang et al.^1^ states in this connection that *“therefore using TPA as standard to measure phorbol esters could not give reliable results” …”..the chemical structures of Jatropha phorbol esters are quite different from that of TPA”*.

The chemical structures of Jatropha PEs and TPA/PMA are definitely different, but this does not mean that TPA cannot be used as external or internal standard. It has been shown in a series of publications that TPA is a reliable standard, but it of course has different response factors, above all with the use of a UV detector because of different UV absorption. Therefore, the values of phorbol esters are mostly described as TPA equivalents.

Furthermore, in a recent study done by Neu et al.^16^, not cited by Wang et al.^1^, isolated phorbol esters were used as external standard with standard addition and the phorbol esters in the oil were unambiguously identified with their MS data. The results correlate with measurements with TPA as standard.

Wang et al.^1^ further claims that *“In this report, no phorbol esters have been obtained from 2000 kg KCakeJ. An isomeric mixture of 11-hydroxy-9E-octadecenoic acid, 12-hydroxy-10E-octadecenoic acid and 12-hydroxy-10Zoctadecenoic acid (hydroxy-octadecenoic acids, molecular formula of the individuals C18H34O3) has been isolated and identified as the main toxic components of KCakeJ.”*

No information is provided regarding the origin of the seeds that were used to produce the Jatropha seed cake or the treatments that these underwent before it was purchased by the authors. Therefore, it is difficult to evaluate this statement. If the cake was solvent extracted multiple number of times, for example, no residual PE will be retained in the cake. Another possibility could be that the seedcake has been obtained from edible provenance of *Jatropha curcas*. In toxic *Jatropha curcas*, phorbol esters have been shown to be present at higher levels in oil compared to seedcake. Analysis of phorbol esters in oil from the seeds used in the study by Wang et al.^1^ would have provided more information, which was not attempted.

In the papers published prior to that of Wang et al.^1^, phorbol esters were conclusively shown to be the toxic principle of Jatropha (other antinutrients including the lectin, curcin, being degraded by heat treatment), whose presence or absence makes Jatropha seed kernels non-edible or edible, respectively. On the other hand, a scan of the literature on *Jatropha curcas* (6500 papers between 1949 and 2021 according to Chemical Abstracts Sci-Finder) shows no report on the occurrence of hydroxy octadecenoic acid derivatives in any plant part of *Jatropha curcas*. In over 10 years of scientific work on Jatropha in laboratories of Prof. Dr Klaus Becker and Prof. Dr Martin Mittelbach, where jatropha samples from all over the world were subjected to chemical analysis, there was no evidence on the existence of hydroxy-octadecenoic acid in the oil or cake. This, however, does not mean that such compounds were not found by Wang et al.^1^, but because inadequate description of the seed cake is provided, the results and the conclusions based on it have to be questioned. The pre-treatment and storage conditions of the Jatropha seed cake used by Wang et al.^1^ are not mentioned in the publication. This information may shed some light on the origin of these compounds.

From literature, it is obvious that there are serious discrepancies between the findings of Wang et al.^1^ and previous scientific findings of other groups. Furthermore, the conclusions of Wang et al.^1^ are not substantiated by the information presented in their work. Key information, such as the source of the seeds from which the seed cake procured was produced, the treatments that the seed cake underwent before procurement and the duration and conditions of storage of the seed cake are missing in the paper. Due to several serious shortcomings, the key conclusions of the paper are not justified.

## Supplementary information

Supplementary information
