# Supplementary material for: Critique on conclusions regarding toxic compounds in Jatropha curcas kernel cake
Source: Commun Biol. 2021 Dec 1;4:1348. doi: 10.1038/s42003-021-02869-6 (PMC8636474; doi:10.1038/s42003-021-02869-6)
Supplement: Supplementary file 1 — Supplementary information [file 42003_2021_2869_MOESM1_ESM.docx]

***To the Author****— Please review the editorial comments and requests below and confirm that changes have been made in the manuscript in the right-hand column. The completed document* ***must be uploaded*** *as a related manuscript file.*

| **Files and General Policies** | |
| --- | --- |
| Main manuscript file must be in Microsoft Word or LaTeX format.  LaTex and Tex article source files must be accompanied by the compiled PDF for reference. The bibliography must be submitted separately (as a .bib file) or contained within the .tex file. | Our manuscript is in MS Word format |
| An updated editorial policy checklist that verifies compliance with all required editorial policies must be completed and uploaded with the revised manuscript. All points on the policy checklist must be addressed; if needed, please revise your manuscript in response to these points.  <https://www.nature.com/documents/nr-editorial-policy-checklist.zip>  Please note that this form is a dynamic ‘smart pdf’ and must therefore be downloaded and completed in Adobe Reader. Clicking this link will download a zip file containing the pdf. | Editorial policy checklist duly complied to |
| **ORCID**  *Communications Biology* is committed to improving transparency in authorship. As part of our efforts in this direction, we are now requesting that all authors identified as ‘corresponding author’ create and link their Open Researcher and Contributor Identifier (ORCID) with their account on the Manuscript Tracking System (MTS) prior to acceptance. ORCID helps the scientific community achieve unambiguous attribution of all scholarly contributions. For more information please visit <http://www.springernature.com/orcid>.  For all corresponding authors listed on the manuscript, please follow the instructions in the link below to link your ORCID to your account on our MTS before submitting the final version of the manuscript. If you do not yet have an ORCID you will be able to create one in minutes.  <https://www.springernature.com/gp/researchers/orcid/orcid-for-nature-research>  IMPORTANT: All authors identified as ‘corresponding author’ on the manuscript must follow these instructions. Non-corresponding authors do not have to link their ORCIDs but are encouraged to do so. Please note that it will not be possible to add/modify ORCIDs at proof. Thus, if they wish to have their ORCID added to the paper they must also follow the above procedure prior to acceptance.  To support ORCID's aims, we only allow a single ORCID identifier to be attached to one account. If you have any issues attaching an ORCID identifier to your MTS account, please contact the Platform Support Helpdesk at <http://platformsupport.nature.com/> | OK |
| **Title Page** | |
| Please ensure that the author list provided in our manuscript tracking system matches the author list in the main manuscript. | OK |
| Please note that titles should be a single declarative sentence of ~15 words. | OK |
| The manuscript should begin with a brief paragraph that summarizes the message of the article without specialized terminology, for a non-specialist readership. | OK |
| **Main text** | |
| The manuscript should be no more than 1200 words, unless previously agreed otherwise with the editor. Please do not include headings or subheadings in the main text. We recommend including a concluding or summary paragraph at the end of the main text. | The size of the manuscript accepted previously and reviewed |
| Please insert the following at the beginning of the main text:  **Arising from Wang, XH., Liu, JQ., Chen, S. et al. Commun Biol 3, 228 (2020), https://doi.org/10.1038/s42003-020-0919-z.** | OK |
| **End Notes** | |
| Please check that your bibliography complies with the following:   - Your bibliography should start with the heading “References”. The references must be numbered in the order of appearance in the text, then tables, then figures. - Any in-text citations to references (e.g. "Gupta et al. show...") should be followed by their corresponding reference citation number from the reference list. - Manuscript citations must include journal title, article title, volume number, page or article number or DOI, and year of publication. - No publication can be present more than once in the reference list. - No footnotes are permitted in the references or elsewhere. Text should be incorporated into the main text, the Methods section, or the Supplementary Information instead. - Websites should only be listed in the references if they are in common use or curated. - Where possible, preprints in the reference list should be updated with details of the published, peer-reviewed paper. - Citations should be formatted in the text using superscript numbers. | OK |
| Please check that your 'Author Contributions' section individually lists the specific contribution of each author to the work. Each author must be referred to by name or initials. Where multiple authors possess identical initials, they must be clearly disambiguated from one another.  See our author contributions policy for further information: <https://www.nature.com/nature-research/editorial-policies/authorship#author-contribution-statements> | OK |
